# A binding-enhanced but enzymatic activity-eliminated human ACE2 efficiently neutralizes SARS-CoV-2 variants

**DOI:** 10.1038/s41392-021-00821-y

**Published:** 2022-01-11

**Authors:** Anqi Zheng, Lili Wu, Renyi Ma, Pu Han, Baoying Huang, Chengpeng Qiao, Qihui Wang, Wenjie Tan, George F. Gao, Pengcheng Han

**Affiliations:** 1 CAS Key Laboratory of Pathogenic Microbiology and Immunology, Institute of Microbiology, Chinese Academy of Sciences, Beijing, 100101 China; 2 University of the Chinese Academy of Sciences, Beijing, 100049 China; 3 National Institute for Viral Disease Control and Prevention, Chinese Center for Disease Control and Prevention (China CDC), Beijing, 102206 China; 4 School of Medicine, Zhongda Hospital, Southeast University, NanJing, 210009 China

**Keywords:** Molecular biology, Structural biology

**Dear Editor**,

The coronavirus disease 2019 (COVID-19) pandemic, caused by severe acute respiratory syndrome coronavirus 2 (SARS-CoV-2), is a great threat to global public health. Although several vaccines and therapeutic antibodies have been authorized for emergency use, several studies have reported that they show weakened protective effects against SARS-CoV-2 variants, including Alpha, Beta, Gamma, and currently dominant Delta and Lambda.^1^ Thus, this has become the biggest challenge for COVID-19 vaccine and therapeutic antibody clinical applications. Studies have shown that soluble angiotensin-converting enzyme 2 (ACE2), the receptor for SARS-CoV-2, can neutralize the virus by acting as a competitor of endogenous ACE2 and thus has potential as a therapeutic protein.^2^ However, pharmacokinetic analysis demonstrated that the half-life of the recombinant ACE2 (rACE2) protein is short, lasting only a few hours.^3^ Other studies showed that a fusion protein consisting of the extracellular domain of human ACE2 (hACE2) fused to the Fc region of human immunoglobulin IgG1 (hACE2-hFc or hACE2-Ig) had long lasting effects, can bind to the receptor binding domain (RBD) of the spike (S) proteins of SARS-CoV-2, and can neutralize SARS-CoV-2 in vitro and in vivo.^4^ Although several hACE2 variants have been engineered to optimize their binding to the S protein of SARS-CoV-2, the understanding about their inhibitory activities against the current SARS-CoV-2 variants are limited. Thus, our goal is to generate a recombinant ACE2 protein that is long lasting without enzymatic activity and can potently neutralize different SARS-CoV-2 variants.

To screen efficient ACE2 fusion proteins with neutralization activity against both the wild-type SARS-CoV-2 strain (SARS-CoV-2 WT) and variants, we designed a panel of hACE2 proteins with mutations in the binding interface with the SARS-CoV-2 RBD. Analysis of the structure of the SARS-CoV-2 RBD/hACE2 complex showed that most residues in hACE2 interacted with SARS-CoV-2 RBD are hydrophobic or charged. We focused on five potentially critical hydrophobic or charged residues, T27, D30, K31, H34, and M82, and designed seven mutants (T27F, D30E, K31R, H34F, H34W, M82F, and M82W) that enhance their hydrophobicity or electrification. During our study, other groups also optimized the above sites to enhance the neutralizing ability of ACE2-Ig.^5,6^ We fused the Fc domain of mouse IgG1 (mFc) to the C-terminus of hACE2 mutants as a tag for subsequent expression and purification. Then, the binding of the hACE2-mFc mutants to the SARS-CoV-2 RBDs was assessed using surface plasmon resonance (SPR). We found that the binding affinities of two hACE2-mFc mutants, T27F and D30E, were enhanced, the affinities of two others, K31R and H34F, were diminished, and the affinities of the remaining three, H34W, M82F, and M82W, were nearly identical to that of wild-type hACE2 (hACE2-WT) (Fig. 1a). Notably, of the two enhanced mutants, T27F showed stronger binding to the SARS-CoV-2 RBD than D30E, which was approximately three times stronger than that of hACE2-WT. Similarly, Li et al. reported D30E mutant neutralized SARS-CoV-2 pseudovirus more efficiently than ACE2-WT, that was consistent with our result.^5^ Of the two diminished mutants, H34F displayed weaker binding to SARS-CoV-2 RBD, which was approximately five times weaker than that of hACE2-WT. As a result, we selected the binding-enhanced hACE2-T27F mutant as the candidate for further study.

**Fig. 1:**
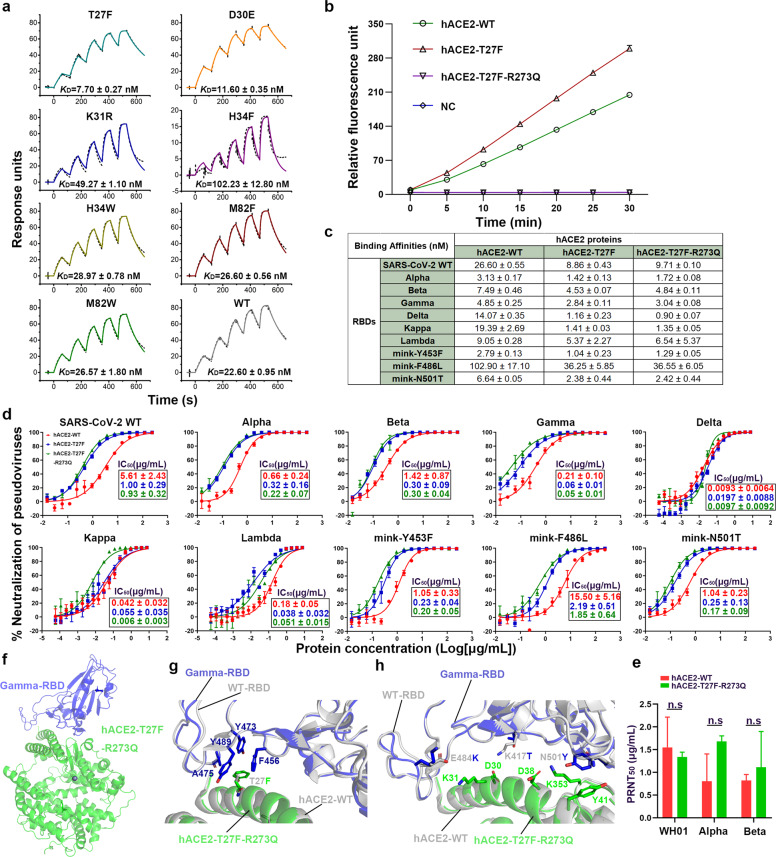
Binding and neutralization activities of an enzymatic activity-eliminated hACE2 mutant against SARS-CoV-2 WT and variants, and the complex structure of it with Gamma-RBD. **a** Soluble mFc-tagged hACE2 mutants were captured by anti-mIgG Fc antibodies immobilized on the CM5 chip. Serially diluted SARS-CoV-2 RBDs were flowed over the chip surface to assess binding to the hACE2 mutants. hACE2-WT was used as a control. The raw and fitted curves are shown as dotted and solid lines, respectively. The binding affinities (*K*_D_) are shown as mean ± SEM of three independent experiments. **b** The enzymatic activity of hACE2-T27F-R273Q was assessed using the ACE2 Activity Fluorometric Assay Kit according to the manufacturer’s instructions. NC, negative control (buffer only). Relative fluorescence values were determined for 30 min at 5 min intervals. Representative data (mean of triplicates, *n* = 3) from three independent experiments are shown, and the bars represent the SD. **c** hFc-tagged hACE2-WT and mutants were captured on protein A chip, and serial dilutions of the RBDs of SARS-CoV-2 WT, Alpha, Beta, Gamma, mink-Y453F, mink-F486L, and mink-N501T were flowed over the chip surface to assess binding to the hACE2 proteins. The *K*_D_ values are summarized in the table (mean ± SEM of three independent experiments), and the binding curves are shown in Fig. S2. **d** GFP-tagged SARS-CoV-2 WT and variants pseudoviruses were incubated with two-fold serial dilutions of hACE2-WT, hACE2-T27F, or hACE2-T27F-R273Q protein. Then, the mixtures were added to Vero cells. After 15 h, the infected cells were counted with a CQ1 Confocal Quantitative Image Cytometer. The experiments were performed thrice with three replicates (*n* = 3) in each experiment. The curves show representative data, and the IC_50_ values are the mean ± SEM of three independent experiments. **e** 50–80 plaque-forming unit (PFU) of authentic WH01, Alpha or Beta were incubated with two-fold serial dilutions of hACE2-WT or hACE2-T27F-R273Q protein. Then, the mixtures were added to Vero cells. After 1 h, the cellular supernatant was discarded with the addition of DMEM medium containing Avicel. After another 72 h, the numbles of plaques were counted. The experiments were performed twice. The 50% plaques reduction neutralization test titers (PRNT_50_) values were represented as mean ± SD with the bar representing the SD value. n.s, not significant (multiple *t* tests). **f** The overall complex structure of hACE2-T27F-R273Q bound to Gamma-RBD. hACE2-T27F-R273Q and Gamma-RBD were colored in green and blue, respectively. **g** The detailed interaction contributed by F27 on hACE2-T27F-R273Q, and the residues participated in the interaction were labeled with a cutoff of 4.5 Å. **h** The detailed interaction contributed by T417, K484, and Y501 on Gamma-RBD, and the residues involved in the interaction were labeled with a cutoff of 4.5 Å

For clinical application as an antiviral against SARS-CoV-2 infection in the future, we changed the mFc-tag of the hACE2-T27F to hFc. Additionally, to reduce the possible side effects of the rACE2, we impaired the enzymatic activity of the hACE2-T27F by introducing an additional mutation at the R273 position (R273Q), which is a key site for enzymatic activity,^7^ generating hACE2-T27F-R273Q mutant. We assessed the enzymatic activity of the hACE2-T27F-R273Q using the ACE2 Activity Fluorometric Assay Kit. As expected, we found that the enzymatic activity of hACE2-T27F-R273Q was nearly eliminated with no difference to that of the negative control (Fig. 1b). Meanwhile, we assessed the half-life of hACE2-hFc in mice, which was much longer than hACE2 (31.3 h vs 10 h) (Supplementary Fig. S1)^3^. These results suggested that the enzymatic activity-eliminated hACE2-T27F-R273Q has the potential to be used as a long lasting and side effect-free antiviral therapeutic.

To investigate the interactions between hACE2-T27F-R273Q and the SARS-CoV-2 WT and variants, we first used SPR to measure the binding affinities of hACE2-T27F-R273Q to the RBDs of SARS-CoV-2 WT; six human variants, Alpha, Beta, Gamma, Delta, Kappa, and Lambda; and three variants spreading in minks, mink-Y453F, mink-F486L, and mink-N501T. We found that the affinities of hACE2-WT for all variant RBDs, except for mink-F486L, were higher than that for SARS-CoV-2 WT, especially for Alpha and mink-Y453F (~8–10 folds higher than SARS-CoV-2 WT; Fig. 1c and Supplementary Fig. S2). The affinity of the RBD of the mink-Y486L variant interacted with hACE2-WT was four times lower than that of SARS-CoV-2 WT. Interestingly, the affinities of hACE2-T27F-R273Q for all RBDs were similar to the affinities of hACE2-T27F, and both were 2–3 times higher, but ~14 times higher for Delta and Kappa RBDs, than that of hACE2-WT.

We further assessed the inhibitory activities of hACE2-T27F-R273Q against pseudotyped and authentic SARS-CoV-2 WT and variants in Vero cells. The pseudotyped SARS-CoV-2 WT and variants were produced using a vesicular stomatitis virus (VSV) pseudotyped virus production system. We found the hACE2-WT and mutant proteins can neutralize both SARS-CoV-2 WT and variants infection with decreased the half maximal inhibitory concentrations (IC_50_) (Fig. 1d). Our data showed that hACE2-WT can effectively neutralize most SARS-CoV-2 variants with 5–600 times higher neutralizing activities than against SARS-CoV-2 WT, and it was particularly effective against Kappa and Delta (Fig. 1d). For mink-F486L variant, the neutralization of hACE2-WT was weaker than against SARS-CoV-2 WT. Notably, the neutralizing activities of hACE2-T27F-R273Q against SARS-CoV-2 WT and the variants were similar to those of hACE2-T27F, and the IC_50_ values were 2–7 times higher than those of hACE2-WT against these strains. These results were consistent with the binding assays (Fig. 1c, d). hACE2-WT neutralized authentic SARS-CoV-2 WT (WH01), Alpha and Beta strains were as good as against these pseudoviruses (Fig. 1e). Although hACE2-T27F-R273Q neutralized the authentic Alpha and Beta were slightly weaker than hACE2-WT, it showed no significant difference on inhibiting both strains and the WH01 (Fig. 1e).

To elucidate the molecular basis of the binding-enhanced hACE2-T27F-R273Q interacting with SARS-CoV-2 RBD, we solved the structure of hACE2-T27F-R273Q in complex with Gamma-RBD at 2.7 Å resolution (Supplementary Table S1). The overall structure resembled our previously determined complex structure of wild-type SARS-CoV-2 RBD (WT-RBD) bound to hACE2-WT with the root mean square deviation (RMSD) of 0.478 Å for 667 Cα atoms (Fig. 1f). Residues contributing to the interaction between hACE2-T27F-R273Q and Gamma-RBD were listed in Supplementary Table S2, which were almost consistent with the WT-RBD/hACE2-WT interaction, with similar contacts. Due to the aromatic side chain of phenylalanine (F), the hACE2 with T27F mutation enhanced the hydrophobic interaction with Gamma-RBD with additional 11 contacts (Fig. 1g and Supplementary Table S2). Compared to WT-RBD, Gamma-RBD with K417T mutation destroyed the salt bridge interactions with D30 on hACE2-T27F-R273Q, as well as with E484K mutation, which abolished the salt bridge interactions with K31, that both mutations lost five contacts in total (Fig. 1h and Supplementary Table S2). Gamma-RBD with N501Y mutation introduced additional 20 contacts to hACE2-T27F-R273Q and added the π-π interaction with Y41, which resulted in the higher binding to hACE2 (Fig. 1h and Supplementary Table S2), that was consistent with the SPR assays (Fig. 1c). When superimposed the two ACE2s in the WT-RBD/hACE2-WT complex and Gamma-RBD/hACE2-T27F-R273Q complex, the RMSD just was 0.359 Å for 489 Cα atoms, which indicated that the hACE2 structure remained stable with R273Q mutation (Supplementary Fig. S3).

The COVID-19 pandemic continues to expand and multiple SARS-CoV-2 variants have emerged. Although various monoclonal neutralizing antibodies have been developed as potential COVID-19 therapeutics, they could induce the resistant variants. Nevertheless, ACE2-Ig as an antiviral can overcome the problem of virus escape mutation, and several ACE2 mutants have been reported with increased neutralization activity against SARS-CoV-2, compared to ACE2-WT, but their inhibitory capability against variants were poorly understood. Here, we generated an optimized, binding-enhanced, and enzymatic activity-eliminated hACE2 mutant (hACE2-T27F-R273Q) fused to the hFc that can effectively neutralize both SARS-CoV-2 WT and several variants. We revealed the molecular mechanisms of binding enhancement of hACE2-T27F-R273Q. Our binding and neutralization analyses indicate that hACE2-T27F-R273Q has potential as a broad antiviral therapeutic against current and future SARS-CoV-2 variants, and provides insights into its potential for the treatment of infections of other SARS-like coronavirus who use the ACE2 as entry receptor.

## Supplementary information

Supplementary Materials

Validation report
