# Supplementary material for: A binding-enhanced but enzymatic activity-eliminated human ACE2 efficiently neutralizes SARS-CoV-2 variants
Source: Signal Transduct Target Ther. 2022 Jan 11;7:10. doi: 10.1038/s41392-021-00821-y (PMC8748180; doi:10.1038/s41392-021-00821-y)
Supplement: Supplementary file 1 — Supplementary Materials [file 41392_2021_821_MOESM1_ESM.docx]

Supplementary Materials for

**A** **binding-enhanced but** **enzymatic activity-eliminated human ACE2 efficiently neutralizes SARS-CoV-2 variants**

**Anqi Zheng, Lili Wu, Renyi Ma, Pu Han, Baoying Huang, Chengpeng Qiao, Qihui Wang, Wenjie Tan, George F. Gao, Pengcheng Han**

Correspondence to: Pengcheng Han: [pengchenghan85@163.com](mailto:pengchenghan85@163.com)

This PDF file includes:

**Materials and Methods**

**Figures S1** Pharmacokinetic of hACE2-hFc in mice

**Figures S2** Binding between hACE2-T27F-R273Q and the RBDs of SARS-CoV-2 WT and variants by SPR

**Figures S3** Structural comparison of hACE2-T27F-R273Q to hACE2-WT

**Tables S1** Data collection and refinement statistics of hACE2-T27F-R273Q in complex with Gamma-RBD

**Tables S2** Comparison of hACE2-T27F-R273Q/Gamma-RBD complex and hACE2-WT/WT-RBD complex.

Materials and Methods

**Gene cloning**

Construction of the pCAGGS-hACE2-mFc plasmid, which expresses the ectodomain of human ACE2 (residues 1–740, accession number: BAJ21180) fused to the Fc domain of mouse IgG1 (mFc) and was used for SPR assays, was described previously^1^. We generated a series of hACE2 mutations in pCAGGS-hACE2-mFc. Then, for clinical application of ACE2 as an antiviral against SARS-CoV-2 infection, we replaced the mFc-tag with the Fc domain of human IgG1 (hFc) and generated plasmids expressing wild-type and mutant hACE2-hFc. In addition, the ectodomain of hACE2-T27F-R273Q (residues 1–615) with a 6×His tag at the C-terminus was synthesized and cloned into the pET21a vector for crystallization.

The coding sequence of the RBD of wild-type SARS-CoV-2 (residues 319–541, GISAID: EPI_ISL_402119) was cloned into pCAGGS with a C-terminal 6×His-tag. Similarly, we generated plasmids expressing the RBDs of Alpha, Beta, Gamma, Delta, Kappa and Lambda and three strains spreading in minks.

**Protein expression and purification**

To prepare mFc-tagged or hFc-tagged hACE2 proteins, pCAGGS-derivative plasmids containing the coding sequences of hACE2 WT or mutants were transiently transfected into HEK293T cells. After 48 h, supernatant containing the indicated proteins was collected, centrifuged, and used for SPR assays.

The RBDs proteins of SARS-CoV-2 WT and variants used for the SPR and crystallization assays were expressed as described for the hACE2 proteins and purified by nickel affinity chromatography using a HisTrap HP 5 mL column (GE Healthcare). The proteins were further purified via gel filtration chromatography using a Superdex 200 column (GE Healthcare) with 20 mM Tris-HCl (pH 8.0) and 150 mM NaCl buffer using the ÄKTA System as described in our previous study ^1^.

The His-tagged hACE2-T27F-R273Q protein was overexpressed as inclusion bodies in *Escherichia coli* strain BL21 (DE3). Then the inclusion bodies were refolded as previously^2^. Briefly, the dissolved inclusion bodies were diluted dropwise in refolding buffer (100 mM Tris-HCl, 400 mM L-Arginine, 2 mM EDTA, 5 mM reduced glutathione, and 0.5 mM oxidized glutathione, pH 8.0) at 4℃ overnight. The refolded protein was purified using a Superdex 200 Increase column (GE Healthcare) in a gel filtration buffer (20 mM Tris, 150 mM NaCl, pH 8.0) for crystallized preparation.

To obtain the hACE2-T27F-R273Q and Gamma-RBD complex, purified hACE2-T27F-R273Q and Gamma-RBD proteins were mixed and incubated for 1 h on ice. The mixture was further purified with a Superdex 200 Increase column (GE Healthcare) in 20 mM Tris-HCl (pH 8.0) and 150 mM NaCl buffer. The complex peak of the hACE2-T27F-R273Q with Gamma-RBD was collected and concentrated to ~10 mg/mL for crystallization.

**SPR analysis**

We determined the binding affinities of each mFc-tagged or hFc-tagged hACE2 proteins with the RBDs of SARS-CoV-2 WT or mutants by SPR using a BIAcore 8K (GE Healthcare) at 25℃ in single-cycle mode. The running buffer contained 20 mM HEPES (pH 7.4), 150 mM NaCl, and 0.005% (v/v) Tween 20, and the RBDs of SARS-CoV-2 WT and mutants were exchanged to this buffer by gel filtration before use. For the mFc-tagged hACE2s, anti-mFc antibodies were first immobilized on the CM5 chip using amine-coupling chemistry protocol (GE Healthcare). For the hFc-tagged hACE2s, a protein A chip was used (GE Healthcare). Then, supernatants containing mFc-tagged or hFc-tagged hACE2s were injected and respectively captured, at approximately 500 response units, by the CM5 or protein A chip. Serially diluted RBDs of SARS-CoV-2 WT or mutants were flowed over the chip surface to assess their binding to the hACE2 proteins. The CM5 and protein A chips were regenerated with 10 mM Glycine-HCl at pH 1.7 and 1.5, respectively. The equilibrium dissociation constant (*K*_D_) of each pair of interaction was analyzed using BIAcore® 8K Evaluation Software (GE Healthcare) by fitting the data to a 1:1 Langmuir binding model.

**Enzymatic activity assay**

We assessed the enzymatic activities of hACE2 proteins using the ACE2 Activity Fluorometric Assay Kit (Beyotime). Briefly, the hACE2 proteins were diluted to 785 ng/mL using the assay buffer in the kit. Then, 10 μL of the hACE2 protein was mixed with 88 μL of assay buffer and reacted with 2 μL of substrate. The relative fluorescence values were determined using a Varioskan LUX (Thermo Scientific) for 30 min at 5 min intervals. The data were analyzed using GraphPad Prism 8.0.

**Pharmacokinetic of hACE2-hFc in mice**

Specific pathogen-free (SPF) BALB/c mice were injected I.P. with 10 mg/kg wild-type hACE2-hFc (3 mice/group), respectively. Serum samples were collected from mice at 0, 5, 24, 72, and 120 h post injection. The concentration of hACE2-hFc protein in serum was determined by enzymatic activity assay. The hACE2-hFc protein diluted into control serum was used to generate a standard curve. The relative fluorescence values were determined using TECAN Infinite 200 PRO. The data were analyzed using PKSolver and GraphPad Prism 8.0.

**Pseudovirus** **neutralization assay**

We produced GFP-tagged SARS-CoV-2 WT and variants pseudoviruses using a vesicular stomatitis virus (VSV) pseudotyped virus production system as previously described^3^. In brief, HEK293T cells expressing the S protein of SARS-CoV-2 WT or variants were infected with the rVSV-△G virus. At 30 h after infection, supernatants containing the indicated pseudovirus were harvested, centrifuged, and stored at -80℃ until use. For the neutralization assay, Vero cells were seeded in 96-well plates at 10^4^ cells/well at 24 h before infection. 50 μL of supernatant containing 1,000 fluorescence focus units (FFU) of pseudovirus was incubated with an equal volume of two-fold serial dilutions of hACE2 proteins for 1 h at 37 °C. The concentration of hACE2 proteins in the mixtures ranged from 0.235 to 240 μg/mL. The mixtures were then added to the Vero cells in triplicate. After 15 h, the infected cells were counted with a CQ1 Confocal Quantitative Image Cytometer (Yokogawa). The half maximal inhibitory concentration (IC_50_) of hACE2 protein was calculated using GraphPad Prism 8.0.

**Authentic virus neutralization assay**

Vero cells were seeded in 12-well plates (1×10^6^ cells/well) and cultured for overnight. 150 μL of supernatant containing 50-80 plaque-forming unit (PFU) of authentic SARS-CoV-2 WT (WH01 strain) or variants (Alpha and Beta strains) was incubated with an equal volume of serially diluted hACE2 proteins for 1 h at 37 °C. The cell culture was removed, 300 uL of the virus-protein mixture was added to each well, and the culture was incubated for 1h. The mixture was removed, 1mL of avicel in DMEM containing 2% FBS was added, and cultured for 3 days. The cells were fixed with 4% paraformaldehyde at room temperature for 30 min, and stained with crystal violet for 5 min. The plaques were counted, and the 50% plaques reduction neutralization test titers (PRNT_50_) of hACE2 protein was calculated using GraphPad Prism 8.0.

**Crystallization, data collection, and structure determination**

The crystallization of the hACE2-T27F-R273Q/Gamma-RBD complex was performed using the vapor-diffusion sitting-drop method, with 0.8 μL protein mixing with 1 μL reservoir solution at 18 °C. High-resolution crystals were obtained at 0.1 M magnesium chloride, 0.1 M MES (pH 6.0) and 8% w/v polyethylene glycol 6000. Diffraction data were collected at the Shanghai Synchrotron Radiation Facility (SSRF) 02U1. The data were indexed, integrated, and scaled with HKL2000^4^. The structure of the hACE2-T27F-R273Q/Gamma-RBD was determined by the molecular replacement method using Phaser^5^ with the previously reported structure of hACE2/SARS-CoV-2 RBD (PDB: 6LZG) as the search model. The atomic models were built with Coot^6^ and refined with Phenix^7^. Data collection, processing, and refinement statistics are summarized in Supplementary Table 1. The structure was analyzed using PyMOL (<https://pymol.org/2/>).

**Reference**

1 Wang, Q. *et al.* Structural and functional basis of SARS-CoV-2 entry by using human ACE2. *Cell* **181**, 1-11 (2020).

2 Wu, L. *et al.* Broad host range of SARS-CoV-2 and the molecular basis for SARS-CoV-2 binding to cat ACE2. *Cell Discov* **6**, 68 (2020).

3 Nie, J. *et al.* Quantification of SARS-CoV-2 neutralizing antibody by a pseudotyped virus-based assay. *Nat. Protoc.* **15**, 3699-3715 (2020).

4 Otwinowski, Z. & Minor, W. Processing of X-ray diffraction data collected in oscillation mode. *Methods Enzymol.* **276**, 307-326 (1997).

5 Read, R. J. Pushing the boundaries of molecular replacement with maximum likelihood. *Acta Crystallogr. D Biol. Crystallogr.* **57**, 1373-1382 (2001).

6 Emsley, P., Lohkamp, B., Scott, W. G. & Cowtan, K. Features and development of coot. *Acta Crystallogr. D Biol. Crystallogr.* **66**, 486-501 (2010).

7 Adams, P. D. *et al.* PHENIX: a comprehensive Python-based system for macromolecular structure solution. *Acta Crystallogr. D Biol. Crystallogr.* **66**, 213-221 (2010).

Figures S1

**Pharmacokinetic of hACE2-hFc in mice**

**
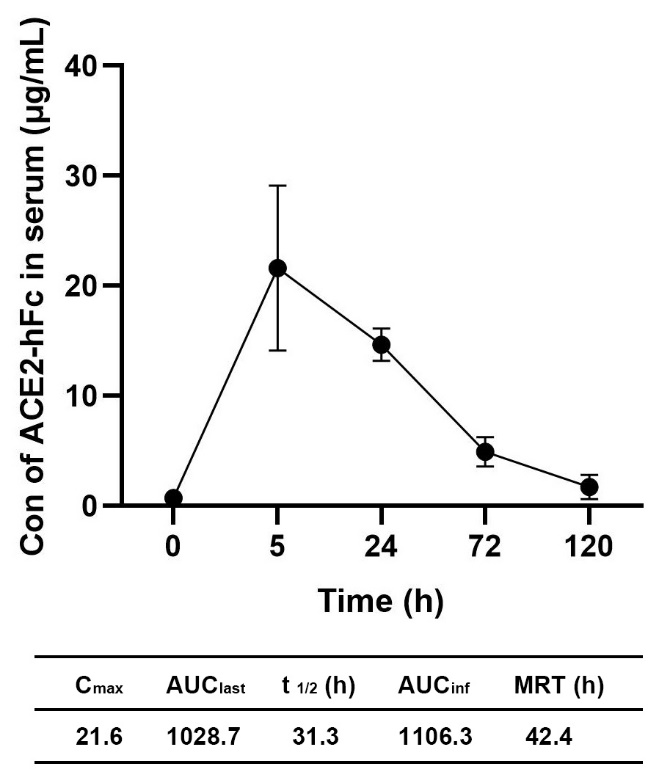
**

Blood samples were collected from mice (n=3) at 0h, 5h, 24h and 72h post administration, and the serum was separated by centrifugation. The concentration of hACE2-hFc in serum was determined by enzyme activity and a standard curve. The data points represented the mean ± SD of three mice，and the pharmacokinetic parameters were analyzed by using PKSolver. C_max_ maximum plasma concentration, AUC_last_ area under the curve, t_1/2_ terminal half-life, AUC_inf_ AUC from time zero to infinity, MRT mean residence time.

Figures S2

**Binding between hACE2-T27F-R273Q and the RBDs of SARS-CoV-2 WT and variants by SPR**


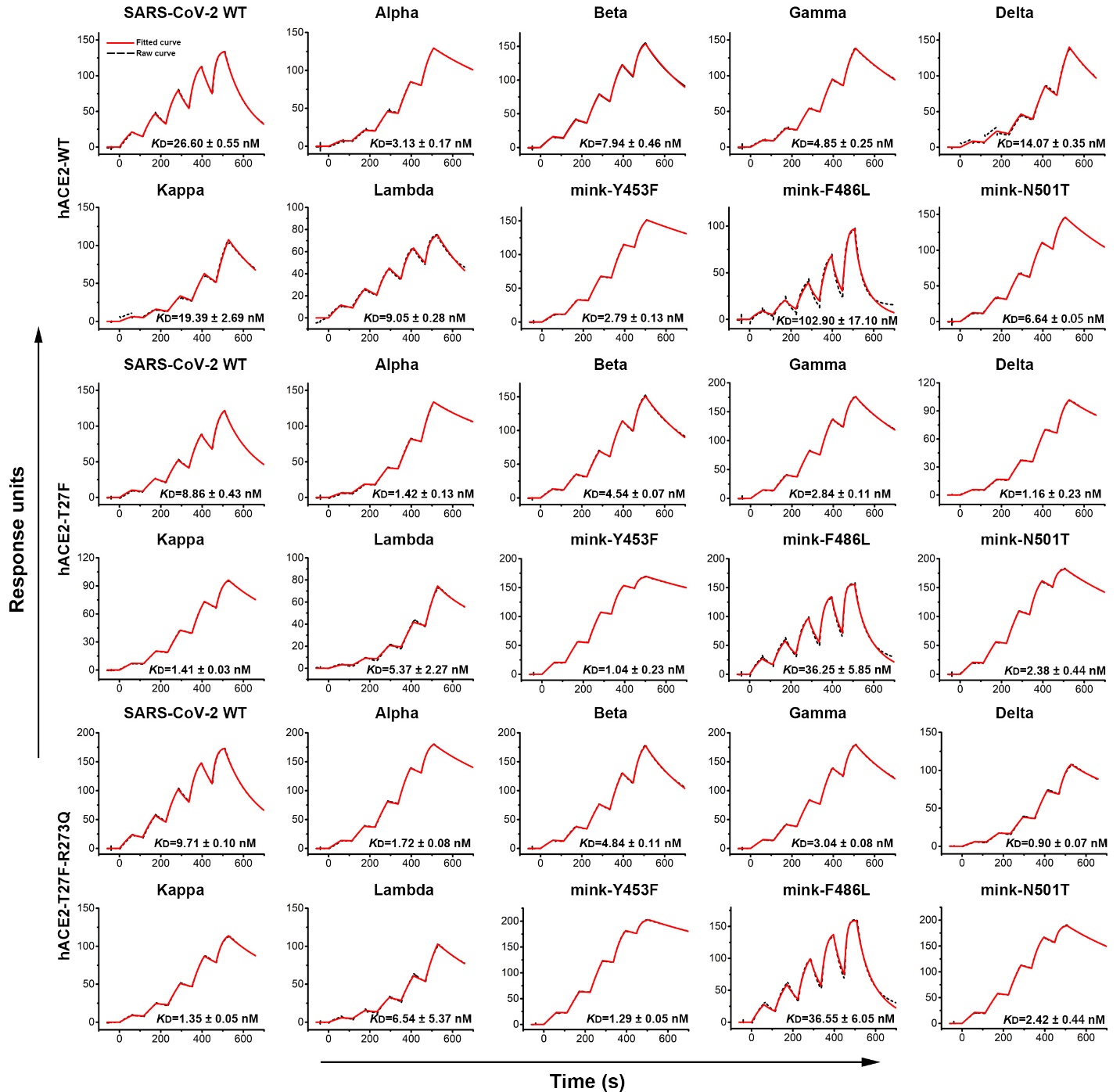


The hFc-tagged hACE2-WT and mutants were captured on protein A chip, and serial dilutions of the RBDs of SARS-CoV-2 WT and variants were then flowed over the chip surface to assess their binding to the hACE2 proteins. The raw and fitted curves are shown as dotted and solid lines, respectively. The binding affinities (*K*_D_) are shown as means ± SEM of three independent experiments.

Figures S3

**Structural comparison of hACE2-T27F-R273Q to hACE2-WT**

**
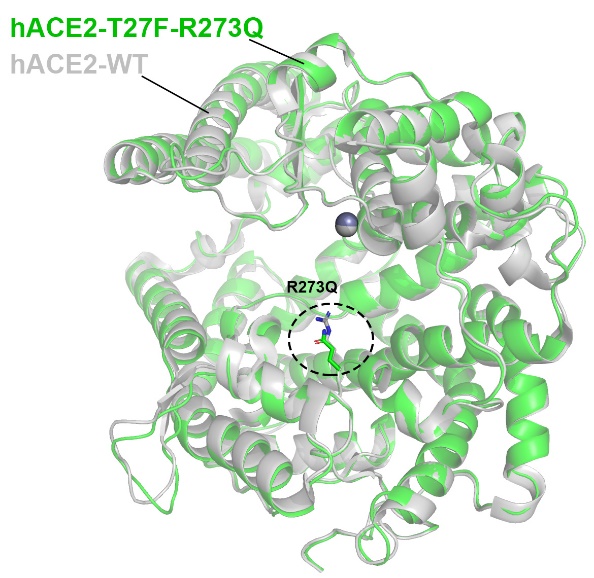
**

The two ACE2s, hACE2-T27F-R273Q and hACE2-WT, extracted from the hACE2-T27F-R273Q/Gamma-RBD complex and hACE2-WT/WT-RBD complex were superimposed. hACE2-T27F-R273Q and hACE2-WT were colored in green and gray, respectively. The R273Q mutation displaying in sticks was circled using dotted ellipse.

Table S1

**Data collection and refinement statistics of hACE2-T27F-R273Q in complex with Gamma-RBD**

|  | hACE2-T27F-R273Q/Gamma-RBD |
| --- | --- |
| **Data collection** |  |
| Space group | P41212 |
| **Cell dimension** |  |
| a, b, c (Å) | 103.35, 103.35, 233.32 |
| α, β, λ (°) | 90.00, 90.00, 90.00 |
| Resolution (Å) | 38.68-2.70 (2.80-2.70) |
| Unique reflections | 35413 (3433) |
| R_merge_ | 0.175 (1.061) |
| R_pim_ | 0.057 (0.362) |
| *I* / σ*I* | 12.73 (1.52) |
| Completeness (%) | 99.8 (98.7) |
| Redundancy | 10.0 (9.3) |
| **Refinement** |  |
| Resolution (Å) | 17.57-2.70 |
| No. reflections | 35406 |
| R_work_/R_free_ | 0.2045/0.2294 |
| No. atoms |  |
| Protein | 6417 |
| Ligands | 1 |
| Water | 104 |
| Average B-factors (Å^2^) |  |
| Protein | 40 |
| Ligands | 84 |
| Water | 40 |
| RMSDs |  |
| Bond lengths (Å) | 0.003 |
| Bond angles (°) | 0.593 |
| Ramachandran plot (%) |  |
| Favored | 97.46 |
| Allowed | 2.41 |
| Outliers | 0.13 |

The highest resolution shell is shown in parentheses.

Table S2

**Comparison of hACE2-T27F-R273Q/****Gamma-RBD complex and hACE2-WT/WT-RBD complex**

| **Gamma-RBD/WT- RBD** | **hACE2-T27F-R273Q** | **hACE2-WT** |
| --- | --- | --- |
| T417/K417 |  | D30 (4,1) |
| V445 | L45 (1) |  |
| G446 | Q42 (3,1) | Q42 (4) |
| Y449 | D38 (8,2), Q42 (3,1) | D38 (9,1), Q42 (4,1) |
| Y453 | H34 (8) | H34 (6) |
| L455 | D30 (2), K31 (2) | D30 (2), K31 (2), H34 (10) |
| F456 | F27 (9), D30 (2), K31 (3) | T27 (5), D30 (4), K31 (5) |
| Y473 | F27 (4) | T27 (1) |
| A475 | S19 (3,1), Q24 (5), F27 (6), | S19 (3,1), Q24 (4), T27 (2) |
| G476 | S19 (4), Q24 (6) | S19 (4), Q24 (5) |
| S477 | S19 (1), Q24 (2) |  |
| K484/E484 |  | K31 (1) |
| F486 | L79 (2), M82 (13), Y83 (13) | L79 (2), M82 (9), Y83 (11) |
| N487 | Q24 (13,1), Y83 (9,1) | Q24 (15,1), Y83 (8,1) |
| Y489 | Q24 (1), F27 (7), F28 (8), K31 (6), Y83 (1,1) | T27 (7), F28 (7), K31 (6), Y83 (1) |
| F490 |  | K31 (2) |
| Q493 | H34 (20), E35 (1) | K31 (3), H34 (6), E35 (11) |
| S494 | H34 (8,1) |  |
| Y495 | K353 (1) |  |
| G496 |  | D38 (2), K353 (5,1) |
| Q498 | Y41 (7), Q42 (5), L45 (2) | D38 (2), Y41 (17), Q42 (12,2), L45 (5) |
| T500 | Y41 (7,1), N330 (8), D355 (9,1), R357 (3) | Y41 (7,1), L45 (1), N330 (8), D355 (8), R357 (3) |
| Y501/N501 | D38 (1), Y41 (13), K353 (25) | Y41 (8,1), K353 (11) |
| G502 | K353 (5,1), G354 (6), D355 (1) | K353 (4,1), G354 (7), D355 (1) |
| Y505 | E37 (3), K353 (23), G354 (4), R393 (1) | E37 (7), K353 (28), G354 (4), R393 (1) |
|  |  |  |
| Total | 298, 12 | 304, 12 |

The numbers in parentheses of RBDs residues represent the number of vdw and H-bond contacts between the indicated residue with hACE2-T27F-R273Q and hACE2-WT. The numbers in parentheses of ACE2s residues represent the numbers of vdw contacts the indicated residues conferred. The numbers behind comma suggest numbers of potential H-bonds between the pairs of residues. vdw contact was analyzed at a cutoff of 4.5 Å and H-bonds at acut off of 3.5 Å. The contacts contributed by residue T27/F27 were shown in red. The contacts contributed by residues K417/T417, E484/K484 and N501/Y501 were shown in blue.
