# Supplementary material for: A binding-enhanced but enzymatic activity-eliminated human ACE2 efficiently neutralizes SARS-CoV-2 variants
Source: Signal Transduct Target Ther. 2022 Jan 11;7:10. doi: 10.1038/s41392-021-00821-y (PMC8748180; doi:10.1038/s41392-021-00821-y)
Supplement: Supplementary file 2 — Validation report [file 41392_2021_821_MOESM2_ESM.pdf]

Preliminary Full wwPDB X-ray Structure Validation Report (i)

Jul 23, 2021 – 06:00 PM JST

Deposition ID : D_1300023460

This is a Preliminary Full wwPDB X-ray Structure Validation Report.

This report is produced by the wwPDB Deposition System during initial deposition but before annotation of the structure.

We welcome your comments at *validation@mail.wwpdb.org*
A user guide is available at
https://www.wwpdb.org/validation/2017/XrayValidationReportHelp
with specific help available everywhere you see the (i) symbol.

The following versions of software and data (see references (i)) were used in the production of this report:

MolProbity	:	4.02b-467
Mogul	:	1.8.5 (274361), CSD as541be (2020)
Xtriage (Phenix)	:	1.13
EDS	:	2.22
Percentile statistics	:	20191225.v01 (using entries in the PDB archive December 25th 2019)
Refmac	:	5.8.0158
CCP4	:	7.0.044 (Gargrove)
Ideal geometry (proteins)	:	Engh & Huber (2001)
Ideal geometry (DNA, RNA)	:	Parkinson et al. (1996)
Validation Pipeline (wwPDB-VP)	:	2.22

1 Overall quality at a glance (i)

The following experimental techniques were used to determine the structure:
X-RAY DIFFRACTION

The reported resolution of this entry is 2.70 Å.

Percentile scores (ranging between 0-100) for global validation metrics of the entry are shown in the following graphic. The table shows the number of entries on which the scores are based.

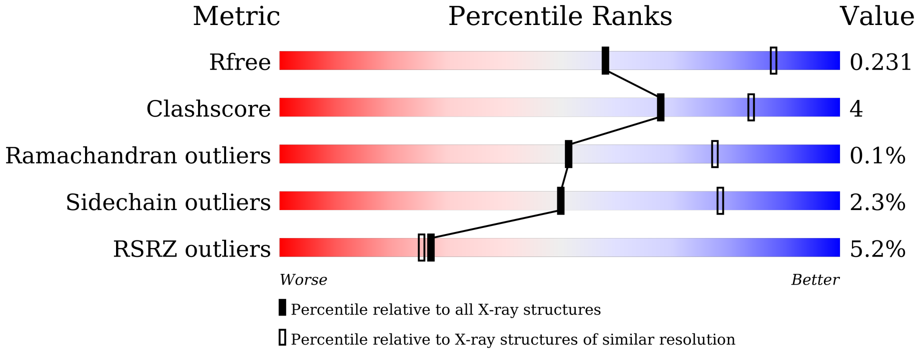

Metric	Whole archive (#Entries)	Similar resolution (#Entries, resolution range(Å))
R_{free}	130704	2808 (2.70-2.70)
Clashscore	141614	3122 (2.70-2.70)
Ramachandran outliers	138981	3069 (2.70-2.70)
Sidechain outliers	138945	3069 (2.70-2.70)
RSRZ outliers	127900	2737 (2.70-2.70)

The table below summarises the geometric issues observed across the polymeric chains and their fit to the electron density. The red, orange, yellow and green segments of the lower bar indicate the fraction of residues that contain outliers for >=3, 2, 1 and 0 types of geometric quality criteria respectively. A grey segment represents the fraction of residues that are not modelled. The numeric value for each fraction is indicated below the corresponding segment, with a dot representing fractions <=5% The upper red bar (where present) indicates the fraction of residues that have poor fit to the electron density. The numeric value is given above the bar.

Mol	Chain	Length	Quality of chain
1	B	596	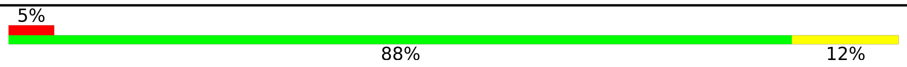
2	B000	194	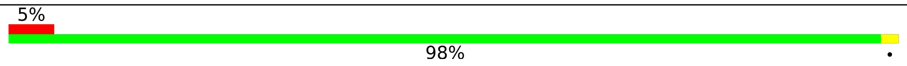

2 Entry composition (i)

There are 5 unique types of molecules in this entry. The entry contains 6522 atoms, of which 0 are hydrogens and 0 are deuteriums.

In the tables below, the ZeroOcc column contains the number of atoms modelled with zero occupancy, the AltConf column contains the number of residues with at least one atom in alternate conformation and the Trace column contains the number of residues modelled with at most 2 atoms.

- Molecule 1 is a protein.

Mol	Chain	Residues	Atoms					ZeroOcc	AltConf	Trace
			Total	C	N	O	S			
1	B	596	4865	3116	803	917	29	0	0	0

- Molecule 2 is a protein.

Mol	Chain	Residues	Atoms					ZeroOcc	AltConf	Trace
			Total	C	N	O	S			
2	B000	194	1538	989	255	286	8	0	0	0

- Molecule 3 is 2-acetamido-2-deoxy-beta-D-glucopyranose (three-letter code: NAG) (formula: $C_8H_{15}NO_6$).

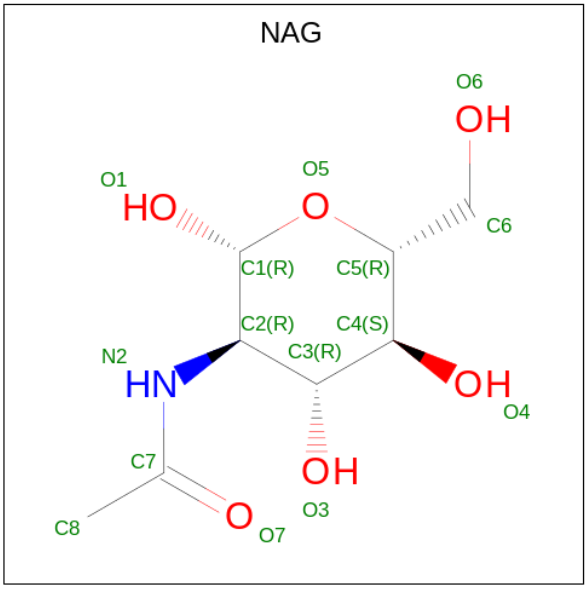

Mol	Chain	Residues	Atoms				ZeroOcc	AltConf
			Total	C	N	O		
3	B000	1	14	8	1	5	0	0

- Molecule 4 is ZINC ION (three-letter code: ZN) (formula: Zn).

Mol	Chain	Residues	Atoms		ZeroOcc	AltConf
			Total	Zn		
4	C	1	1	1	0	0

- Molecule 5 is water.

Mol	Chain	Residues	Atoms		ZeroOcc	AltConf
			Total	O		
5	S	104	104	104	0	0

3 Residue-property plots (i)

These plots are drawn for all protein, RNA, DNA and oligosaccharide chains in the entry. The first graphic for a chain summarises the proportions of the various outlier classes displayed in the second graphic. The second graphic shows the sequence view annotated by issues in geometry and electron density. Residues are color-coded according to the number of geometric quality criteria for which they contain at least one outlier: green = 0, yellow = 1, orange = 2 and red = 3 or more. A red dot above a residue indicates a poor fit to the electron density (RSRZ > 2). Stretches of 2 or more consecutive residues without any outlier are shown as a green connector. Residues present in the sample, but not in the model, are shown in grey.

- Molecule 1:

Chain B:

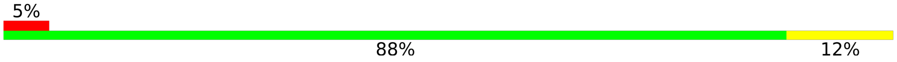

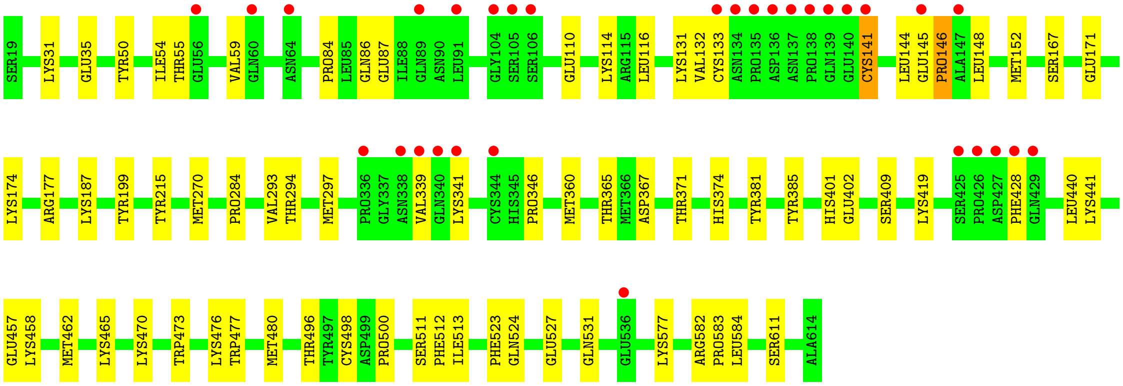

- Molecule 2:

Chain B000:

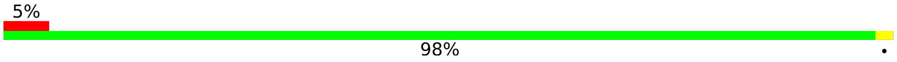

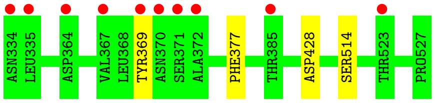

4 Data and refinement statistics (i)

Property	Value	Source
Space group	P 41 21 2	Depositor
Cell constants<br>a, b, c, α, β, γ	103.35Å 103.35Å 233.32Å<br>90.00° 90.00° 90.00°	Depositor
Resolution (Å)	17.57 – 2.70<br>38.68 – 2.70	Depositor<br>EDS
% Data completeness<br>(in resolution range)	99.9 (17.57-2.70)<br>94.5 (38.68-2.70)	Depositor<br>EDS
R_{merge}	(Not available)	Depositor
R_{sym}	(Not available)	Depositor
$< I/\sigma(I) >$ [1]	1.52 (at 2.69Å)	Xtriage
Refinement program	phenix.refine 1.19rc3_4028, PHENIX 1.19rc3_4028	Depositor
R, R_{free}	0.204 , 0.230<br>0.206 , 0.231	Depositor<br>DCC
R_{free} test set	2000 reflections (5.63%)	wwPDB-VP
Wilson B-factor (Å^2)	35.3	Xtriage
Anisotropy	0.406	Xtriage
Bulk solvent k_{sol}(e/Å^3), B_{sol}(Å^2)	0.34 , 37.7	EDS
L-test for twinning[2]	$< \|L\| >$ = 0.48, $< L^2 >$ = 0.32	Xtriage
Estimated twinning fraction	No twinning to report.	Xtriage
F_o,F_c correlation	0.92	EDS
Total number of atoms	6522	wwPDB-VP
Average B, all atoms (Å^2)	43.0	wwPDB-VP

Xtriage's analysis on translational NCS is as follows: *The largest off-origin peak in the Patterson function is 3.78% of the height of the origin peak. No significant pseudotranslation is detected.*

[1]Intensities estimated from amplitudes.

[2]Theoretical values of $< |L| >$, $< L^2 >$ for acentric reflections are 0.5, 0.333 respectively for untwinned datasets, and 0.375, 0.2 for perfectly twinned datasets.

5 Model quality (i)

5.1 Standard geometry (i)

Bond lengths and bond angles in the following residue types are not validated in this section: NAG, ZN

The Z score for a bond length (or angle) is the number of standard deviations the observed value is removed from the expected value. A bond length (or angle) with $|Z| > 5$ is considered an outlier worth inspection. RMSZ is the root-mean-square of all Z scores of the bond lengths (or angles).

Mol	Chain	Bond lengths		Bond angles	
		RMSZ	#|Z| >5	RMSZ	#|Z| >5
1	B	0.27	0/5003	0.44	0/6798
2	B000	0.28	0/1583	0.49	0/2156
All	All	0.27	0/6586	0.45	0/8954

There are no bond length outliers.

There are no bond angle outliers.

There are no chirality outliers.

There are no planarity outliers.

5.2 Too-close contacts (i)

In the following table, the Non-H and H(model) columns list the number of non-hydrogen atoms and hydrogen atoms in the chain respectively. The H(added) column lists the number of hydrogen atoms added and optimized by MolProbity. The Clashes column lists the number of clashes within the asymmetric unit, whereas Symm-Clashes lists symmetry-related clashes.

Mol	Chain	Non-H	H(model)	H(added)	Clashes	Symm-Clashes
1	B	4865	0	4638	35	0
2	B000	1538	0	0	0	0
3	B000	14	0	0	0	0
4	C	1	0	0	0	0
5	S	104	0	0	0	0
All	All	6522	0	4638	35	0

The all-atom clashscore is defined as the number of clashes found per 1000 atoms (including hydrogen atoms). The all-atom clashscore for this structure is 4.

All (35) close contacts within the same asymmetric unit are listed below, sorted by their clash magnitude.

Atom-1	Atom-2	Interatomic distance (Å)	Clash overlap (Å)
1:B:284:PRO:HD3	1:B:440:LEU:HD22	1.79	0.63
1:B:457:GLU:HG2	1:B:512:PHE:HB3	1.82	0.61
1:B:458:LYS:O	1:B:462:MET:HG3	2.02	0.60
1:B:527:GLU:O	1:B:531:GLN:HG3	2.05	0.57
1:B:116:LEU:HD21	1:B:187:LYS:HD3	1.86	0.57
1:B:187:LYS:HE3	1:B:199:TYR:CZ	2.41	0.55
1:B:144:LEU:HA	1:B:148:LEU:HB2	1.91	0.53
1:B:31:LYS:O	1:B:35:GLU:HG3	2.09	0.52
1:B:524:GLN:HG2	1:B:583:PRO:HG2	1.91	0.52
1:B:419:LYS:HE2	1:B:428:PHE:HB3	1.91	0.51
1:B:152:MET:HG3	1:B:270:MET:HA	1.93	0.51
1:B:367:ASP:O	1:B:371:THR:HG23	2.12	0.50
1:B:215:TYR:CE1	1:B:577:LYS:HD3	2.47	0.49
1:B:132:VAL:HG22	1:B:171:GLU:HG3	1.95	0.49
1:B:470:LYS:HA	1:B:473:TRP:CD1	2.48	0.48
1:B:110:GLU:O	1:B:114:LYS:HG3	2.15	0.47
1:B:31:LYS:HE2	1:B:35:GLU:OE2	2.15	0.47
1:B:50:TYR:CE1	1:B:59:VAL:HG22	2.51	0.46
1:B:174:LYS:HE2	1:B:496:THR:OG1	2.16	0.45
1:B:145:GLU:HB2	1:B:146:PRO:HD3	1.99	0.45
1:B:167:SER:O	1:B:171:GLU:HG2	2.17	0.45
1:B:55:THR:O	1:B:59:VAL:HG23	2.17	0.44
1:B:177:ARG:HB2	1:B:498:CYS:HB2	2.00	0.44
1:B:465:LYS:HE3	1:B:465:LYS:HB2	1.69	0.43
1:B:131:LYS:HD2	1:B:141:CYS:HB3	2.00	0.43
1:B:523:PHE:CD2	1:B:584:LEU:HD12	2.55	0.42
1:B:477:TRP:CE3	1:B:500:PRO:HG3	2.55	0.41
1:B:54:ILE:HD12	1:B:341:LYS:HG3	2.01	0.41
1:B:374:HIS:CE1	1:B:402:GLU:OE1	2.74	0.41
1:B:346:PRO:HB3	1:B:360:MET:HG3	2.03	0.41
1:B:476:LYS:O	1:B:480:MET:HG3	2.21	0.41
1:B:513:ILE:HD12	1:B:513:ILE:HA	1.88	0.41
1:B:294:THR:HG23	1:B:365:THR:HA	2.02	0.41
1:B:293:VAL:O	1:B:297:MET:HG3	2.21	0.40
1:B:84:PRO:HB2	1:B:87:GLU:HG3	2.03	0.40

There are no symmetry-related clashes.

5.3 Torsion angles (i)

5.3.1 Protein backbone (i)

In the following table, the Percentiles column shows the percent Ramachandran outliers of the chain as a percentile score with respect to all X-ray entries followed by that with respect to entries of similar resolution.

The Analysed column shows the number of residues for which the backbone conformation was analysed, and the total number of residues.

Mol	Chain	Analysed	Favoured	Allowed	Outliers	Percentiles	
1	B	594/596 (100%)	586 (99%)	7 (1%)	1 (0%)	47	73
2	B000	192/194 (99%)	180 (94%)	12 (6%)	0	100	100
All	All	786/790 (100%)	766 (98%)	19 (2%)	1 (0%)	51	78

All (1) Ramachandran outliers are listed below:

Mol	Chain	Res	Type
1	B	146	PRO

5.3.2 Protein sidechains (i)

In the following table, the Percentiles column shows the percent sidechain outliers of the chain as a percentile score with respect to all X-ray entries followed by that with respect to entries of similar resolution.

The Analysed column shows the number of residues for which the sidechain conformation was analysed, and the total number of residues.

Mol	Chain	Analysed	Rotameric	Outliers	Percentiles	
1	B	526/526 (100%)	514 (98%)	12 (2%)	50	78
2	B000	167/167 (100%)	163 (98%)	4 (2%)	49	77
All	All	693/693 (100%)	677 (98%)	16 (2%)	50	78

All (16) residues with a non-rotameric sidechain are listed below:

Mol	Chain	Res	Type
1	B	86	GLN
1	B	133	CYS
1	B	141	CYS
1	B	339	VAL
1	B	381	TYR

Continued on next page...

Continued from previous page...

Mol	Chain	Res	Type
1	B	385	TYR
1	B	401	HIS
1	B	409	SER
1	B	441	LYS
1	B	511	SER
1	B	582	ARG
1	B	611	SER
2	B000	369	TYR
2	B000	377	PHE
2	B000	428	ASP
2	B000	514	SER

Sometimes sidechains can be flipped to improve hydrogen bonding and reduce clashes. All (4) such sidechains are listed below:

Mol	Chain	Res	Type
1	B	102	GLN
1	B	394	ASN
1	B	531	GLN
1	B	586	ASN

5.3.3 RNA ⓘ

There are no RNA molecules in this entry.

5.4 Non-standard residues in protein, DNA, RNA chains ⓘ

There are no non-standard protein/DNA/RNA residues in this entry.

5.5 Carbohydrates ⓘ

There are no monosaccharides in this entry.

5.6 Ligand geometry ⓘ

Of 2 ligands modelled in this entry, 1 is monoatomic - leaving 1 for Mogul analysis.

In the following table, the Counts columns list the number of bonds (or angles) for which Mogul statistics could be retrieved, the number of bonds (or angles) that are observed in the model and the number of bonds (or angles) that are defined in the Chemical Component Dictionary. The

Link column lists molecule types, if any, to which the group is linked. The Z score for a bond length (or angle) is the number of standard deviations the observed value is removed from the expected value. A bond length (or angle) with $|Z| > 2$ is considered an outlier worth inspection. RMSZ is the root-mean-square of all Z scores of the bond lengths (or angles).

Mol	Type	Chain	Res	Link	Bond lengths			Bond angles		
					Counts	RMSZ	$\#\|Z\| > 2$	Counts	RMSZ	$\#\|Z\| > 2$
3	NAG	B000	604	2	14,14,15	0.23	0	17,19,21	0.50	0

In the following table, the Chirals column lists the number of chiral outliers, the number of chiral centers analysed, the number of these observed in the model and the number defined in the Chemical Component Dictionary. Similar counts are reported in the Torsion and Rings columns. '-' means no outliers of that kind were identified.

Mol	Type	Chain	Res	Link	Chirals	Torsions	Rings
3	NAG	B000	604	2	-	0/6/23/26	0/1/1/1

There are no bond length outliers.

There are no bond angle outliers.

There are no chirality outliers.

There are no torsion outliers.

There are no ring outliers.

No monomer is involved in short contacts.

5.7 Other polymers (i)

There are no such residues in this entry.

5.8 Polymer linkage issues (i)

There are no chain breaks in this entry.

6 Fit of model and data (i)

6.1 Protein, DNA and RNA chains (i)

In the following table, the column labelled '#RSRZ> 2' contains the number (and percentage) of RSRZ outliers, followed by percent RSRZ outliers for the chain as percentile scores relative to all X-ray entries and entries of similar resolution. The OWAB column contains the minimum, median, 95th percentile and maximum values of the occupancy-weighted average B-factor per residue. The column labelled 'Q< 0.9' lists the number of (and percentage) of residues with an average occupancy less than 0.9.

Mol	Chain	Analysed	<RSRZ>	#RSRZ>2			OWAB(Å^2)	Q<0.9
1	B	596/596 (100%)	0.01	31 (5%)	27	25	24, 40, 63, 99	0
2	B000	194/194 (100%)	0.02	10 (5%)	27	25	28, 40, 88, 99	0
All	All	790/790 (100%)	0.01	41 (5%)	27	25	24, 40, 78, 99	0

All (41) RSRZ outliers are listed below:

Mol	Chain	Res	Type	RSRZ
1	B	339	VAL	5.8
1	B	138	PRO	5.7
2	B000	371	SER	4.9
2	B000	372	ALA	4.9
1	B	139	GLN	4.4
1	B	338	ASN	4.1
1	B	427	ASP	4.0
1	B	136	ASP	3.8
1	B	344	CYS	3.8
1	B	429	GLN	3.8
1	B	428	PHE	3.5
2	B000	334	ASN	3.5
2	B000	370	ASN	3.4
1	B	426	PRO	3.4
1	B	134	ASN	3.3
1	B	140	GLU	3.3
2	B000	367	VAL	3.2
1	B	104	GLY	3.2
1	B	135	PRO	3.1
1	B	133	CYS	3.1
1	B	60	GLN	3.0
2	B000	335	LEU	3.0
1	B	141	CYS	2.9
1	B	89	GLN	2.8

Continued on next page...

Continued from previous page...

Mol	Chain	Res	Type	RSRZ
1	B	56	GLU	2.8
2	B000	523	THR	2.8
1	B	425	SER	2.7
2	B000	369	TYR	2.6
1	B	106	SER	2.5
1	B	64	ASN	2.4
1	B	536	GLU	2.4
1	B	336	PRO	2.3
1	B	340	GLN	2.3
1	B	137	ASN	2.3
2	B000	385	THR	2.2
1	B	145	GLU	2.2
1	B	147	ALA	2.2
1	B	91	LEU	2.1
2	B000	364	ASP	2.1
1	B	341	LYS	2.1
1	B	105	SER	2.0

6.2 Non-standard residues in protein, DNA, RNA chains (i)

There are no non-standard protein/DNA/RNA residues in this entry.

6.3 Carbohydrates (i)

There are no monosaccharides in this entry.

6.4 Ligands (i)

In the following table, the Atoms column lists the number of modelled atoms in the group and the number defined in the chemical component dictionary. The B-factors column lists the minimum, median, 95th percentile and maximum values of B factors of atoms in the group. The column labelled 'Q< 0.9' lists the number of atoms with occupancy less than 0.9.

Mol	Type	Chain	Res	Atoms	RSCC	RSR	B-factors($Å^2$)	Q<0.9
3	NAG	B000	604	14/?	0.82	0.32	69,84,92,99	0
4	ZN	C	1	1/?	0.95	0.11	40,40,40,40	0

6.5 Other polymers (i)

There are no such residues in this entry.
